# Beyond the emergency: Centering mental health in community disaster preparedness and recovery

**DOI:** 10.1371/journal.pone.0345216

**Published:** 2026-04-03

**Authors:** Omolola E. Adepoju, Ashley M. Smith, Laura A. de la Roche, David Curtis

**Affiliations:** 1 Tilman J Fertitta Family College of Medicine, University of Houston, Houston Texas, United States of America; 2 Humana Integrated Health Systems Sciences Institute, University of Houston, Houston, Texas, United States of America; University of Luzon, PHILIPPINES

## Abstract

**Background:**

Extreme weather events are increasing in frequency and severity, disproportionately affecting marginalized and under‑resourced communities. In Houston, Texas, where insurance coverage and mental health access remain limited, successive disasters compound existing inequities, leaving residents with unmet psychological needs. Yet little is known about how chronically impacted communities conceptualize their mental health experiences, barriers to accessing care, and priorities for support following repeated disaster events.

**Methods:**

We conducted a qualitative study using town hall–style community conversations in three historically underserved Houston communities (Kashmere Gardens, Third Ward, Fifth Ward). A total of 145 residents participated between March and October 2024. Discussions followed a semi‑structured guide and were captured through trained note‑takers using a standardized template, supplemented by graphic recording. Reflexive thematic analysis, employing an inductive approach and an audit‑trailed consensus process, was used to identify themes. Researcher triangulation, persistent observation, and reflexivity were used to enhance trustworthiness.

**Results:**

Three themes emerged. (1) Living in “survival mode”: residents described cumulative emotional exhaustion driven by repeated disasters, including persistent anxiety, hypervigilance, grief, caregiving burdens, and limited capacity for self‑care. (2) Navigating stigma, mistrust, and limited access: participants highlighted cultural stigma, confusing and costly pathways to care, and mistrust stemming from unfulfilled institutional promises and inaccessible services. (3) Building a path forward: residents emphasized the need for mental health literacy, community‑embedded services, improved communication with city officials, and sustained funding for affordable, accessible, and culturally responsive mental health support.

**Conclusions:**

Successive disasters create chronic psychological strain in high‑vulnerability communities while simultaneously limiting access to care. Findings underscore the need for disaster mental health strategies that move beyond the acute response period to include community‑embedded supports, continuity of care, and investments in mental health literacy and affordability. Centering community voices is critical for developing equitable, sustainable, and culturally attuned disaster mental health systems.

## Introduction

Mental health issues affect over one-fifth of the general population, but disproportionately affects minority and lower income populations [1]. In these communities, understanding of mental health is often framed within the context of concerns, problems, or disorders [2]. This reactive conceptualization, the blurring of mental health and mental illness, contributes to stigmatization [3] and overlooks ways in which individuals, communities, and systems can promote mental health and wellness. While changing this awareness can be inherently challenging, it can be even more difficult when individuals are experiencing distress, particularly in the wake of successive climate-related disasters [4]. Experiences of grief and loss, emotional reactivity to the disaster, and financial, housing and food insecurity, often supersedes the ability to attend to psychological symptoms.

In Houston, Texas— the state with the highest uninsurance rate in the nation, with rates almost two-times the national average— individuals from marginalized communities often do not have the resources, or easy access to traditional mental health resources [5]. In the wake of disaster events, such as the recent Hurricane Beryl, the city’s healthcare resources are further strained, with facilities closures arising from flood or wind damage, lack of power, or healthcare workers unable to safely commute to healthcare facilities. These individual-, provider- and system-level effects make it harder for individuals to access the mental health support that they may need. As such, limited access to mental health care in the aftermath of repeated disasters may delay or prevent recovery, causing symptoms to worsen over time.

Further complicating this is the increasing frequency of disaster events. Every subsequent hurricane, or flood, adds another layer of hardship, preventing full recovery and leaving the community in a constant state of vulnerability and distress. The repeated cycle of trauma, loss, and instability brought about by successive disasters can have a profound impact on mental health, leading to chronic distress, a sense of hopelessness, and long-term psychological effects depending on how harrowing residents’ lived experiences were through those disaster events [6]. Others have described the provision of basic (e.g., housing, food, transportation) [7] and mental health resources in the days and weeks immediately following the disaster event (known as the “honeymoon phase”) during which there is higher community cohesion [8]. However, communities have reported a higher need for continued mental health resources, months after disasters hit, after the honeymoon phase.

To address this gap, we designed a study to explore the lived experiences of community members from three historically underserved communities in Houston, Texas, in the aftermath of successive climate-related disaster events. Studies of this nature are important because mental health patterns in disadvantaged populations remains under-researched, making it crucial to understand their experiences, the mental health resources available and utilized, and their unmet needs following disaster events. Insights gained from this study will contribute to the existing literature and inform policies and funding priorities to better support these vulnerable communities.

## Methods

### Participants

Participants included members from three historically underserved communities within the Houston metropolitan area: Kashmere Gardens, Third Ward, Fifth Ward. All three communities were chosen because of the prominent proportions of non-Hispanic African American (AA) residents: Greater Third Ward (68%), Greater Fifth Ward (48%), and Kashmere Gardens (67%). All three communities have Social Vulnerability Index (SVI) rankings in the 80th percentile, representing the highest vulnerability to disaster events in the Houston Area [9–11]. Additionally, these three communities are historically well-established African-American communities where the research team have previously worked.

### Procedure

The authors worked with trusted community leaders from the three communities to organize town-hall style community conversations at culturally significant community locations within each community. Recruitment efforts included social media, email announcements and word-of-mouth outreach at long-standing community meetings and events which took place from the beginning of March to the end of October of 2024. The community conversations began with a group gathering to establish the purpose and objectives of the meeting, fostering a shared understanding of the goals. Following the introductory session, participants broke into smaller roundtable discussions to encourage more focused and interactive dialogue. Each roundtable included 8–10 community residents and was guided by a trained facilitator. A dedicated note-taker, independent of the facilitator, supported each roundtable to ensure that all voices were heard and that the discussions were accurately documented.

Because audio recording was not permitted due to community leaders’ preference and to promote a psychologically safe environment, we ensured rigor and reliability by using a structured note-taking process. All note-takers used a standardized template developed by the research team to document major themes, emerging subthemes, group consensus points, and verbatim participant statements. Prior to data collection, all note-takers completed a brief training on accurate and unbiased note-taking, procedures for identifying and capturing direct quotes in real time, and methods for minimizing interpretation during initial documentation.

During each session, note-takers documented verbatim comments, alongside contextual observations. Immediately following the session, facilitators and note-takers met to review, compare, and reconcile their notes to ensure completeness and consistency across tables. This structured approach enhanced the credibility and dependability of the qualitative data despite the absence of audio recordings.

During the roundtable discussions, we sought to achieve what Morgan et al. (2014) referred to as Real Talk, a term that is commonly used in the African American community which means that a person is talking candidly and honestly about their feelings without fear or reservations of what others might think. Creating a safe and open space for candid expression was a priority, as it allowed participants to share their perspectives authentically and without inhibition. Accordingly, no recordings of the group discussions were taken to increase participant comfort and trust.

**Fig 1:**
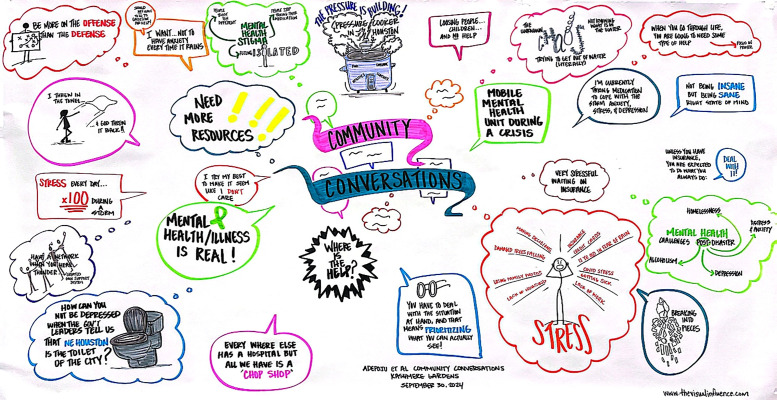
Graphic recording from one of the community events.

Additionally, we incorporated graphic recording as an arts-based engagement approach. In this method, a professional artist visually captured participants’ ideas and expressions in real time, creating a dynamic and interactive record of the discussions. The use of graphic recording encouraged participants to clarify and refine their thoughts and made the research process more tangible, approachable, and engaging. This visual representation of the dialogue not only enriched the discussions but also provided participants with an immediate sense of ownership and validation of their contributions. Graphic recordings from this study are presented in Fig 1.

Participants were not provided financial compensation for attending the townhall sessions; however, each attendee received a disaster preparedness kit containing approximately $100 worth of essential supplies to ensure participant well-being, mental health support was embedded directly into the town hall structure. A clinical psychologist, a psychiatrist, the Chief Behavioral Health Officer from the University’s FQHC–look-alike clinic, and two licensed mental health counselors were present at each session. These professionals offered on-site support, provided brief counseling as needed, and were available to respond to participants who disclosed ongoing distress. Participants expressing continued mental health challenges were connected to these providers, and follow-up appointments were arranged when appropriate to facilitate access to longer-term care.

This study was approved by the University of Houston Institutional Review Board (IRB #00004432). Given the town hall-style format of the community conversations and the complete anonymization of all collected data, the IRB determined that the study met the criteria for exemption and waived the requirement for informed consent. This waiver was granted in accordance with federal regulations, as the study posed minimal risk to participants and did not involve the collection of identifiable private information. Although written consent was waived, participants were informed about the purpose of the study, their voluntary participation, and their right to withdraw at any time. Verbal consent was obtained and documented at the start of each conversation.

### Measures

A semi-structured interview guide was established to direct the group discussions. Questions remained open-ended and inquired about the individuals’ experiences related to mental health following a natural disaster. For example, groups were asked ‘How did you mentally handle or process these natural disasters as they happened?” and “What kind of support or resources would you like to see before, during, or after an event?”. Facilitators allowed group discussions to build between group members and guided the discussion back to remain focused on the question presented when necessary.

### Data analysis

Reflexive thematic analysis was employed to analyze the notes taken from each community conversation event. We followed updated guidelines for conducting reflexive thematic analysis six-phase process to engage with, review, and analyze the data prior to writing the final report [12]. A core analytic team of four researchers (two faculty investigators, one postdoctoral research fellow, and one trained research assistant) participated in all phases of the analysis. In Phase 1 (familiarization), all four team members independently reviewed the consolidated field notes from each table. In Phase 2 (initial coding), the same four analysts generated preliminary codes using an inductive approach. In Phase 3 (theme development), the team met weekly to compare coding decisions, resolve discrepancies through consensus, and cluster related codes into candidate themes. In Phase 4 (reviewing themes), the team refined and collapsed themes across data sources to ensure internal coherence and distinctiveness. In Phase 5 (defining and naming themes), the analysts collaboratively developed clear operational definitions and finalized theme labels. In Phase 6 (reporting), the faculty investigators led the synthesis of findings, with all team members contributing to the selection of illustrative quotations and the refinement of the thematic narrative.

To enhance consistency and rigor, analytic memos were maintained throughout the process, and all analytic decisions were documented in a shared audit trail. An inductive approach was maintained through the entirety of the data analysis process to ensure the voices of the community were accurately represented [13,14].

### Trustworthiness

Multiple methods of trustworthiness were employed to ensure scientific rigor [15]. Specifically, we implemented researcher triangulation [16], persistent observation, and reflexivity [17,18]. Numerous researchers across disciplines were involved in the study from conceptualization and met frequently to critically discuss the analytical process. Finally, reflexivity is critical to ensure credibility of qualitative research; this involves active researcher reflection on their own experiences and preconceptions that may influence the findings. Reflexivity was discussed across authors throughout data analysis.

## Results

A total of 145 community residents participated in the town-hall style community conversations, as shown in Table 1. In Kashmere Gardens, 70.6% of participants were Black (48/68), 23.5% were Hispanic (16/68), and 5.9% were White (4/68). In Third Ward, 68.2% were Black (15/22), 18.2% were Hispanic (4/22), and 13.6% were White (3/22). In Fifth Ward, 60.0% were Black (33/55), 36.4% were Hispanic (20/55), and 3.6% were White (2/55). Females represented the majority in each neighborhood: 67.6% in Kashmere Gardens (46/68), 63.6% in Third Ward (14/22), and 76.4% in Fifth Ward (42/55).

**Table 1:** Demographic details of the attendees at the community conversation events.

	Kashmere Gardens (n = 68)	Third Ward (n = 22)	Fifth Ward (n = 55)
Race/Ethnicity			
Black	48	15	33
Hispanic	16	4	20
White	4	3	2
Biological Sex			
Female	46	14	42
Male	22	8	13

The data generated three themes: the effects of a natural disaster on the mental health of underserved communities; understanding and addressing the barriers to mental health support uptake; and enabling change. Quotes taken by notetakers are included in quotation marks. No demographic or participant details are assigned to quotes to maintain anonymity.

### Theme 1. Living in “Survival mode”: The cumulative emotional toll of caregiving, survival pressures, and ongoing environmental stressors

The first theme includes discussions related to the mental health status of both the community and the individual community members. Participants described how they manage their mental health and attempts made to support the mental health of fellow community members. Finally, participant-identified negative outcomes attributed to poor mental health following disaster-related events.

#### Fear that lingers: Persistent anxiety and hypervigilance *after* each storm.

Participants attributed family loss, negative and overwhelming emotions, and the development of mental health disorders (e.g., depression; anxiety) directly to the occurrence of a natural disaster. One participant described the impact of the disaster on their mental health as; “when the storm hit, I felt my mental health chip away”. The severity of the negative mental health outcomes and high “stress” due to the natural disaster was described as being too overwhelming to manage. Feelings of hopelessness in the weeks following a natural disaster were further identified; “during the two weeks of disaster, my husband was very depressed, and all our hard work felt destroyed”.

Negative outcomes reinforced fear of future disasters, exacerbating concerns about their potential impact on mental health. Participants expressed this sentiment, stating, “We may recover from [the hurricane], but the fear is still there. For example, one parent identified how her “son gets very anxious every time it rains” and another stated that “our son prefers that we keep the plywood over the window” following the hurricane. Results indicate a perception by participants that most individuals who lived within the three underserved communities during a hurricane now live with post-traumatic stress; “everyone in this room has some sort of PTSD”. The continued impact of natural disasters on the wellbeing and lives of participants were further reinforced through discussions of family loss and associated feelings of survivor’s guilt.

#### “No time *to* think *about* myself”: Limited Capacity *for* self-care while *in* survival mode.

Participants discussed their ability, or lack thereof, to manage their own mental health concerns. Participants described being in ‘survival mode’, where their mental health concerns were too overwhelming to be able to identify, focus on, or manage. Groups discussions identified a lack of time and capacity to care for oneself; “there’s no time, not even to think about yourself”.

The importance and use of spiritual and/or religious outlets were identified as helping individuals cope. These outlets provided a sense of comfort, hope, and strength during difficult times, allowing individuals to navigate their mental health struggles more effectively. Activities such as prayer, meditation, and seeking guidance from faith leaders were identified as essential tools for maintaining emotional balance and resilience. Finally, avoidance strategies were described; many participants articulated how they avoided thinking about the disaster event, or “distract[ed] [themselves] with work”.

While most identified they could not access mental health support and services, some participants had professional psychological services set up previously that continued care. These participants described how their need had increased due to the hurricane and their “therapist had to increase my dosage because my stress was so severe”. A need for “therapy for my kids to help deal with trauma” was identified and associated with the natural disaster. Groups discussions embodied a perception of pushing through the challenges and hard times associated with natural disasters. Participants described resiliency despite experiencing mental health struggles and “hav[ing] to keep starting over – stopping isn’t an option. Storms keep coming but I’m adapting”. Despite demonstrating strong resilience and determination to overcome challenges and manage their mental health, participants still reported significant difficulties in achieving these goals.

#### The weight *of* being “Strong”: Caregiving burdens and masking personal distress.

Participants identified the importance of taking care of others in their community, especially family members. Close family and friend relationships to support their mental health were deemed critical to coping. The provision of social support was further identified as beneficial with bi-directional positive effects; “sometimes I talk with my friends face-to-face, it helps, often I had to get up and help so I wouldn’t feel helpless”.

Conversely, supporting others was described by some to come at a cost, with participants highlighting the challenge of prioritizing their own well-being while caring for others. Many felt compelled to present a brave face, saying, “It’s hard to think of yourself when you have the responsibility of helping others” and “talking to family and showing them that I’m put together.” The mental health of others frequently took precedence, leaving participants concerned about their own needs: “I feel like I need to take care of my disabled son and sick sister first. People call me strong, but I am not—I just push. Now that my sister is gone, I think about who will take care of me.” The burden of supporting family and community emerged as a central theme in their experiences.

#### Community conditions that deepen or buffer distress.

Negative community mental health outcomes associated with natural disasters were identified. Increased crime rates instigated fear in participants, who described how “after storms robbing and looting” occur, and storm damages increase participant concern as “with the broken fence in our neighborhood, we’re worried about thieves getting through”. Though participants felt safe in their home, this feeling of security did not extend beyond that; “in our home we are safe but occasionally we hear gunshots and sometimes see blood in the street”. The negative mental health effects following natural disasters were also associated by some participants with increased rates of homelessness within their community.

### Theme 2: Navigating a maze of stigma, mistrust, and limited access—Why Mental health support remains out of reach

Theme two included discussions regarding the barriers experienced related to accessing mental health support services. Groups discussions highlighted numerous barriers participants experienced and identified within their community to seeking out and/or obtaining mental health support. Individual, cultural, and community barriers to mental health service uptake are discussed, as well as participant identified needs within the community to increase the use of mental health services by community members.

#### “People think you’re crazy”: Cultural and community stigma *as a* silencing force.

A salient concern was the continued presence of stigma related to mental health and seeking out support. Stigma was described to come from one’s family, community, and culture. While participants argued that mental health and the use of services “needs to be normalized”, normalization was not their reality. Culture and the community were further identified as contributing to the stigmatization of mental health. Participants expressed a fear of community stigma, noting that seeking mental health support was still considered “taboo” and that “there’s a stigma about going to the doctor or even a psychiatrist—people think you’re crazy.” This stigma serves as a significant barrier, not only to accessing mental health resources but also to openly discussing mental health challenges.

#### “I don’t even know where *to* start”: Limited knowledge and confusing pathways *to* care.

Participants identified a lack of community knowledge regarding mental health resources. Methods of access for mental health resources was further unclear: “It’s difficult to know where to start if you don’t have informational resources”. Participants felt mental health support should be provided by city officials, and clear paths of access outlined for community members. Group discussion demonstrated how many individuals felt that using mental health support services was not feasible due to cost; “mental health is an economic issue too, its expensive to take care of so we don’t take care of it, but we think about it”. The lack of available and affordable mental health care was therefore a barrier to access and uptake; “unless you had some sort of insurance, you do what you always do – deal with it”.

#### “*A* lot *of* promises not fulfilled”: Mistrust *of* providers and institutions.

Uptake of mental health resources was further hindered by service location and accessibility barriers. Services that were available were located too far from the community, and not everyone was comfortable with virtual care to explore that option. One participant described how they “have a psychiatrist who gives medicine, but my psychologist had to move offices, and I can’t do zoom”. Engagement with staff of mental health services was described to be a barrier to access, as participants describe “being hung up on from [staff] of mental health resource [centers]”. These experiences added to general feelings of distrust towards mental health professionals by the community. Specifically, distrust in mental health professionals was clear, with participants expressing concerns that “the [mental health] help… can make it worse.” This distrust extended beyond professionals to the broader provision of mental health services, fueled by “a lot of promises not fulfilled.” Participants described a history of unfulfilled commitments from government officials to provide mental health support, leaving their communities without the promised resources.

### Theme 3: Building a path forward – Strengthening mental health systems through improved awareness, community-based access, and sustained investment

The final theme included discussions related to changes that could, or should, be made to increase the accessibility and uptake of mental health support services. Implications were discussed regarding mental health literacy education, steps towards getting mental health resources within the community, and how financial concerns and funding is tied to mental health within these disadvantaged communities.

#### “We need more information”: Strengthening mental health literacy *to* support early recognition and help-seeking.

A lack of mental health literacy within the community was identified despite its perceived importance. Participants described how members of their community do not seek out mental health resources when they should due to limited self-awareness of mental health challenges. Participants were clear in stating, “we need more information” and “we need to increase education”. Group discussion highlighted a need for additional education and awareness regarding mental health and steps to obtain associated resources within the community.

#### Bringing services closer *to* home: integrating mental health support directly *into the* community.

Discussions identified the paucity of mental health service locations and practitioners within disadvantaged communities. The community also expressed frustration that local policy makers and city officials were not listening to the needs of the disadvantaged communities within Houston. While support was given to other neighborhoods and locations, their community was being left with no support, as one participant stated, “something has to burst into flames for us to get attended to”. Participants felt as though public scrutiny was necessary to incite positive action in the provision of mental health support to their communities. Participants highlighted the need for an organized line of communication between communities and the city.

#### Investing in what matters: Funding affordability, recovery, and long-term mental health *care.*

Participants voiced frustration, concern, and anger regarding the perceived lack of funding being directed towards mental health support and disaster recovery from national and federal entities. Financial strain was identified as a direct cause of poor mental health. Participants attributed feelings of anxiety, stress, and depression to their financial status that had resulted from a disaster event and articulated how “we need free mental health, or reduced cost-options”. Due to the impact of financial barriers on mental health service access, participants identified a need for specific funding directed towards the provision of mental health care for their communities, specifically following natural disasters.

## Discussion

In this study examining the impact of successive climate-related disasters in underserved Gulf Coast communities, findings demonstrate a substantial perceived negative impact on mental health and well-being. By highlighting cumulative trauma, persistent barriers to care, and community-driven solutions, these findings extend existing disaster literature by centering lived experiences in chronically impacted, structurally disadvantaged neighborhoods—an understudied context that reveals how repeated exposures and systemic inequities jointly shape mental health outcomes and service uptake. This perspective offers unique, place-based insights to inform more responsive and equitable post-disaster mental health interventions.

Concerns regarding individual-level disaster preparation most commonly included access to basic necessities, preparing a course of action for the next disaster, and ability to physically prepare for a disaster. These concerns align with current literature, which suggests socioeconomically disadvantaged people to be less prepared for disasters than their counterparts in regard to essential goods (e.g., water, food, medication) [19,20], courses of action (e.g., made a plan, signed up for alerts), and more costly preventative actions (e.g., having flood insurance, ‘rainy day’ savings, home maintenance) [21]. Having unmet basic needs, which may be magnified in the face of a disaster event serves as a predictor for adverse mental health outcomes and mortality [6], highlighting the importance of increasing personal-preparedness efforts within the community.

Various coping strategies were identified to self-manage mental health including spiritual resources and social support (e.g., family/friends, community) after a disaster event. Work by Osofsky et al. expands upon these findings, highlighting the ability of spirituality as a means to cope and church groups as a form of immediate community and mutual aid [22]. Our findings indicating the importance of family/friend and community support and connectedness among community align with previous research. Given that studies indicate community engagement can provide positive mental health experiences following natural disasters [23–25], these behaviors have become key components of communal coping [26] and post-traumatic growth [24,25].

Our findings also highlight concerns about coping with the loss of family members, and processing post-disaster emotions. These concerns align with work by Zareiyan et al. documenting an increased prevalence of prolonged grief disorder (PGD) following disasters [27]. Poor mental health outcomes further align with experienced recorded following a 2018 flood, where residents reported stress, physical and mental exhaustion, and grief [28]. Fear surrounding future disasters aligned with work by Ryu and Fan [29], where participants similarly expressed obsessive thinking, flashbacks, and fear every time it rained after a flood. Ultimately, these experienced symptoms (e.g., emotional instability, stress reactions, and trauma) and the eventual development of mental health conditions (e.g., PTSD, anxiety, depression) have been linked to the impact of disaster events [30].

In the process of living through successive disaster events [31], feeling disregarded or neglected by public leaders and institutions [32] further compounds feelings of helplessness and hopelessness [33]. Poor local government infrastructure [34] and inconsistencies with disaster and emergency management communications were highlighted as key contributors to a sense of helplessness and hopelessness. Isolative behaviors and low mood were described as consequences of feeling left out of important resource communications. For many of these Houston-area residents, discrepancies in crisis responses and resource allocations between impoverished neighborhoods and higher SES communities further reinforced feelings of neglect and discrimination [35].

A need for greater community-level disaster preparation was also identified among participants, with the desire for increased community engagement and organized management. These desires align with aspects of ‘community resilience’ [36–38], a rising mindset in approaching disaster efforts that highlights trust, collaboration [39,40], and involvement of community members in the development and execution of both pre-disaster and recovery preparation efforts [41,42]. This in turn may allow for more accurate identification of disaster preparedness within the community, of necessary resources post-disaster, and coordination with government aid such as FEMA, which has historically faced response and performance issues among work on disaster survivors [43].

Finally, there was a recognition that disasters also present opportunities to enhance mental health literacy. The demand for mental health education and the emphasis on its importance align with findings by McFarlane and Williams, who highlight that disasters can serve as pivotal moments to raise awareness about mental health conditions in communities and underscore the critical role of mental healthcare services [44]. These opportunities may also address the lack of awareness about existing mental health challenges, as individuals with diagnosed disorders reported experiencing heightened symptoms following a disaster [45]. Given the connection between poor mental health outcomes, low mental health literacy [46], and repeated exposure to disasters [47], it is key to incorporate mental health education in disaster planning.

### Enhancing mental health access in underserved communities facing successive disasters

In light of these findings, we propose a community-informed lens to guide mental health preparedness and response efforts during disaster events. Traditional counseling and psychotherapies may not be relevant offerings when community members are experiencing more acute and reactive distress. Within our structured community conversations, residents stated a need for mental health providers to be brought to them as intentionally as “…the Red Cross brings fruit and blankets.” Numerous studies have shown the benefits of providing Mental Health First Aid (MHFA) [48] to promote mental health literacy [49], helping others in distress [50], seeking help for one’s own mental health [51], and reducing stigma [52–55] associated with seeking mental health care [56].

Additionally, public service announcements that encourage community members to utilize mental health resources may come across as ambiguous and potentially stigmatizing. Instead, our efforts should first be directed to informing and educating communities about the services that will be made available over time, illustrating *what* each service consists of, the needs it will address, and the specific outcomes intended. This necessitates a rethinking of the types of services, appropriate doses, and ways to maximize accessibility to care, to ensure appropriate mental health resources at the right time. Mechanisms for continuity of care are then needed to help transition individuals to more conventional models of evaluation, counseling, and pharmacotherapies to then engage in symptom management and wellness promotion.

**Fig 2:**
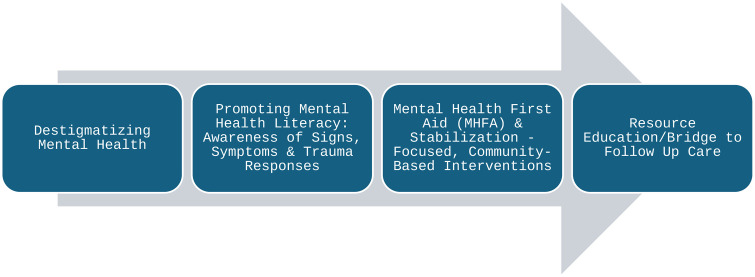
Enhancing mental health access in underserved communities facing successive disasters.

Fig 2 outlines the hypothesized process through which mental health access can be strengthened in underserved communities experiencing successive disasters. Destigmatizing common responses to disasters and modeling discussions about risks and protective factors for subsequent clinical concerns lays the foundation for improving mental health literacy. This sets the stage to help community members better recognize certain signs and symptoms as well as a lexicon for communicating with others about their needs for support. Additionally, immediately following a disaster, it is more important to provide stabilizing supports like MHFA [48] to help community members recognize one another’s needs to better care for each other. Because attending to basic needs and caring for family or neighbors often takes precedence, individuals may delay seeking help for their own concerns [57]. Centering care on both the individual and their role within a broader community system affirms their values and enhances trust. As acute care needs during disasters stabilize, efforts should shift towards establishing mechanisms for bridging emergent care supports to more conventional mental health services. Persistent community-based support extending beyond the initial crisis period will then enable better access to diagnostic evaluations, consultations, and ongoing psychotherapies, and psychiatric medication treatments, when needed.

In conclusion, addressing community mental health during and after disaster events is paramount to ensuring recovery and resilience. With the growing frequency of disaster events, prioritizing mental health preparedness and response in disaster-prone communities is no longer optional—it’s essential.
